# Patterns of sensory and hedonic responses for salty and umami tastes and their impact on food familiarity, consumption, and nutritional status: A gender-based analysis from a large population sample

**DOI:** 10.1016/j.crfs.2025.100970

**Published:** 2025-01-06

**Authors:** Camilla Cattaneo, Sara Spinelli, Caterina Dinnella, Cristina Proserpio, Erminio Monteleone, Ella Pagliarini, Monica Laureati

**Affiliations:** a Sensory & Consumer Science Lab (SCS_Lab), Department of Food, Environmental and Nutritional Sciences (DeFENS), University of Milan, Italy; b SensoryLab, Department of Agriculture, Food, Environment and Forestry (DAGRI), University of Florence, Italy

**Keywords:** Umami, Salty, Savory, Sensory-liking segmentation, BMI, Eating habits

## Abstract

In recent years, research on taste perception has increasingly focused on its influence on food consumption, preferences, and long-term health. While bitter and sweet tastes have been well-studied, less is known about salty and umami tastes and their effects on dietary habits. This study aimed to address this gap by exploring sensory-hedonic patterns for ‘savory’ stimuli, encompassing both umami and salty tastes, in a representative sample of Italian adults, with a focus on gender-specific differences. Associations among sensory-hedonic patterns, nutritional status, personality, and psycho-attitudinal traits, as well as food habits, were considered.

Participants (n = 2878) rated their liking and intensity of salty, umami, and overall flavor sensations for bean purée with varying salt levels and provided anthropometric and food consumption data. K-means clustering identified specific sensory-hedonic patterns: ‘Dislikers’ and ‘Moderate Likers’ in women, and ‘Dislikers’ and ‘Likers’ in men. In both genders, the increased concentration of NaCl in the model food translated in opposite hedonic reactions, which was less evident in men with ‘Likers’ showing a higher preference for the saltiest sample. An overweight condition also characterized this latter group. Both 'Likers' clusters (regardless of gender) showed higher familiarity/consumption of less healthy foods, including high-calorie items, junk foods, meat, and fats (all p < 0.05). Gender-related differences were observed, with women preferring seafood and desserts, while men savory snacks and soft drinks. These results underscore taste's influence on dietary habits and the need to account for gender differences in personalized dietary interventions.

## Highlights

• Study explores sensory-hedonic patterns for salty and umami tastes in Italian adults.
•Two different sensory-hedonic patterns were identified both in women and men.
•Distinct gender-related patterns impact food choices and nutritional status.
•'Likers' of both genders reported higher intake of less healthy food categories.
•Findings emphasize considering gender in personalized dietary interventions.

## Graphical abstract

**Figure:**
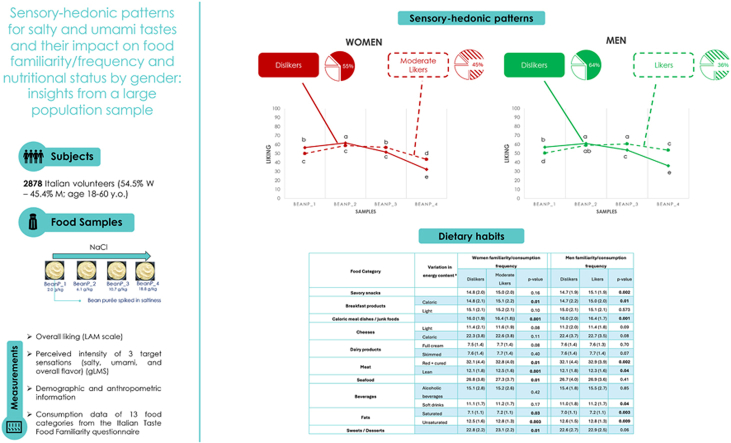

## 1 Introduction

Suboptimal diet quality has been recognized as a leading modifiable contributor to the development and progression of chronic diseases, shaping individuals' susceptibility to conditions such as obesity, type 2 diabetes, and cardiovascular disease (Schulze et al., 2018). However, the intricate interplay between dietary patterns and chronic disease risk is multifaceted and influenced by several factors (Schulze et al., 2018). Thus, elucidating the individual-level determinants of consumption behavior has become imperative in the last decades.

In this scenario, applied taste research has seen a notable shift towards exploring the pivotal role that taste perception plays in shaping food consumption behaviors and, thus, long-term health outcomes. Early studies by Fischer et al. in the 1960s suggested that phenotypic variation in oral sensation influences taste preferences (Fischer et al., 1963). Bitter and sweet taste perceptions have received particular focus, and specific phenotypes have been identified: 6-n-propylthiouracil (PROP) status (Bartoshuk, 1993; Tepper, 2008) and sweet-liking phenotypes (Pangborn, 1970; Iatridi et al., 2019). These phenotypes differently affect food acceptance, eating habits, and nutritional status (Tepper, 2008; Iatridi et al., 2019; Garcia-et al., 2009; Feeney et al., 2011; Armitage et al., 2024; Tan and Tucker, 2019).

Recently, Andres-Hernando et al. (2021) have proposed that compounds eliciting salty and umami (i.e., savoriness-enhancing stimuli) might exacerbate the risk for obesity and metabolic syndrome, although the precise mechanisms remain incompletely understood. Likewise, prior studies have demonstrated a positive correlation between higher BMI and increased preference for or intake of monosodium glutamate (Pepino et al., 2010; He et al., 2008) and sodium chloride (Li et al., 2018). However, less is understood about taste phenotypes or sensory-hedonic patterns related to salty and umami perception and their implications for dietary habits and health. To mention, Lim et al. (2020), investigated differences in psycho-hedonic responses to sweet and umami tastes and their association with energy intake, the proportion of energy from macronutrients, and body composition. However, the segmentation was only based on liking for sweet taste and was conducted involving a limited sample size of female subjects (n = 69). As well, Kubota et al. (2018), tested the effect of umami taste acuity on dietary choices in healthy subjects. In this case, the authors considered only the acuity to umami taste to categorize 44 young female subjects in umami normal- and hypo-tasters. Recently, Endrizzi et al. (2021), performed a large-scale consumer segmentation on the Italian population (n = 2258), grouping subjects into four different clusters based on the correlation between liking and saltiness responsiveness in a model food (bean purée). However, the authors did not consider umami responsiveness in the clustering model applied, and the potential implications of the identified salty taste clusters on actual food consumption. Also, in a preliminary analysis of a smaller cohort (about 400 Italian individuals), Proserpio et al. (2025) identified two clusters differing in salt liking considering only the hedonic response to the bean purée model food and found that salt likers preferred the saltier and fatter options in pairs of different foods, but also the fattest/sweetest option in non-savory products, pointing to a connection between salt liking and unhealthier food choices.

Besides sensory factors, numerous individual variables influence preferences for, and sensitivity to, specific tastes, including gender and personality traits. Gender-related differences in sensory-hedonic patterns have been widely reported (Endrizzi et al., 2021; Hayes et al., 2010; Spinelli et al., 2021; Puputti et al., 2019), as evident in the difference in dietary intake and eating behaviors (Li et al., 2012; Leblanc et al., 2015; Yahia et al., 2016). Women tend to consume more fruits, vegetables, and whole foods, but also indulge in sweets and cakes, while men favor fat and protein-rich foods along with alcoholic and sweetened beverages, potentially predisposing them to overweight and obesity (Deglaire et al., 2015). Women choose less often than men for fat-rich meat (Spinelli et al., 2020) but in both sexes, a higher liking for meat was associated with a higher BMI (Dinnella et al., 2023).

Psychological traits, such as food neophobia, sensitivity to disgust, and sensitivity to reward, were previously investigated in different taste phenotypes (Spinelli et al., 2021; Proserpio et al., 2018; Bajec and Pickering, 2010) and their role in affecting eating behaviors has been reported, suggesting a positive association between food neophobia and responsiveness to ‘warning sensations’ (Laureati et al., 2018; Spinelli et al., 2018) and a negative association with the liking of many food items, especially those that are strong-tasting and unfamiliar or novel in both children and adolescents and adults (Karaağaç and Bellikci-Koyu, 2023). Consistently, adults with higher sensitivity to reward showed a preference for sweet taste (Richardson and Saliba, 2011) and spicy foods (Spinelli et al., 2018; Byrnes and Hayes, 2013). Not only preference for sweet food but also intake of high-energy products is linked to reward sensitivity (De Cock et al., 2016a, 2016b; De Decker et al., 2016). Thus, it is possible to hypothesize that individuals who exhibit higher sensitivity to reward might be expected to align with specific sensory-hedonic patterns more linked to the rewarding properties of palatable foods. However, the majority of studies (e.g. Sutton et al., 2022) focused on the role of fatty and sweet palatable foods and less is understood about the association with personal traits, such as reward sensitivity, and savory taste stimuli or related sensory-hedonic patterns, though savory foods can be innately rewarding (Tsuru et al., 2018).

In the present study, we aimed to examine sensory-hedonic patterns for ‘savory stimuli’, encompassing both umami and salty tastes, in a representative sample of adult Italian consumers. We hypothesized that different patterns could shape consumption habits, thereby impacting overall dietary habits and, consequently, health outcomes. Here, savory-tastes patterns were obtained considering the liking/perceived intensity of these two stimuli in a model food (bean purée) characterized by an umami taste and modified with increasing amounts of added sodium chloride. This approach offers greater ecological validity than previous studies that utilized aqueous solutions (Lim et al., 2020; Kubota et al., 2018) and builds upon the method used by Endrizzi et al. (2021), which focused solely on liking and the perception of saltiness.

Associations among sensory-hedonic patterns, nutritional status, and food consumption habits were evaluated. Given the potential connection between personality traits, psychological characteristics, taste perception, and preferences—particularly responsiveness to salty and umami tastes—food neophobia and sensitivity to reward and punishment were also considered. A within-gender approach was adopted to highlight gender-specific differences.

## 2 Materials and methods

**Fig. 1:**
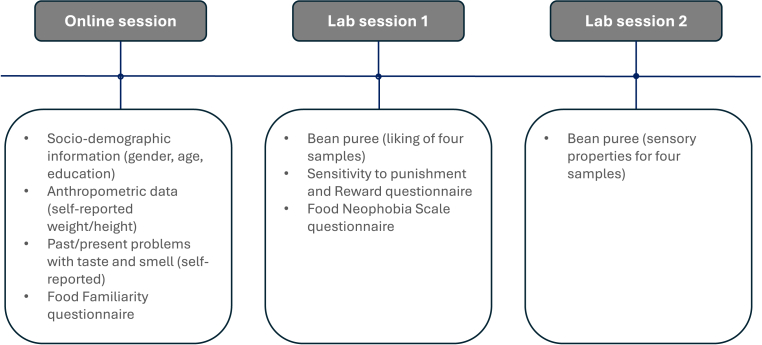
Overview of the study design.

Data presented in this paper were part of the *Italian Taste* project, a large-scale study aimed at investigating, with a multidimensional approach, the influences of biological, genetic, socio-cultural, psychological, and personality-related aspects on food choice and preferences in a large population sample, and their relevance in determining individual differences within a given food culture framework. The complete overview of the study and procedures are described in Monteleone et al. (2017). Upon recruitment, participants received an overview of the study's objectives. They were requested to fill out an online questionnaire from their homes in the days leading up to the data collection. Additionally, they were invited to participate in two sessions, held on separate days, in a sensory laboratory to collect data on liking and perceived intensity and fill out several questionnaires. In the present paper, the following data were considered: demographic, anthropometric, and general health information; familiarity with a series of food categories; overall liking and perceived intensity ratings of bean purée samples spiked with sodium chloride; sensitivity to punishment and reward and food neophobia (please refer to Fig. 1 and the following sections for further details).

### 2.1 Participants

Data were collected on over 3000 Italian subjects recruited on a national basis during the years 2015–2017. The eligibility criteria comprised individuals aged 18–60 years, who were either born in Italy or had resided in Italy for at least 20 years. Participants were selected from key geographical regions across Italy (19 sensory labs (Monteleone et al., 2017)).

Participants were recruited through various methods including internet announcements on the Italian Sensory Science Society website (www.scienzesensoriali.it), the project's website, social media platforms, magazines, emails, pamphlets, and word of mouth. The study was conducted in agreement with the Italian ethical requirements on research activities and personal data protection (D.L. 30.6.03 n. 196). The study protocol was approved by the Ethics Committee of Trieste University (n. 64, June 9, 2015) where the genetic unit of the project was based. The respondents gave their written informed consent at the beginning of the test according to the principles of the Declaration of Helsinki.

### 2.2 Food model

A semisolid food model, easy to prepare, preserve, and serve, was developed using commercially available base ingredients. Bean purée (BeanP) was selected as the appropriate food matrix to elicit the target sensation of saltiness and umami (Monteleone et al., 2017). The bean purée base was made by blending a commercial purée powder mix (Pfanni, Unilever, Italy) with 250 g of canned cannellini beans (Cannellini al vapore, Bonduelle Italy, S.p.A, Italy) and 100 ml of water. Four samples were created by adding increasing concentrations of sodium chloride (BeanP_1: 2.0, BeanP_2: 6.1, BeanP_3: 10.7, and BeanP_4: 18.8 g/kg) to the base formulation. These concentration levels were chosen to produce a range of increased intensity perception of the salty sensation, from weak to strong, also enhancing the umami perception, due to their well-documented synergistic effect (Dunteman and Lee, 2023; Atsumi et al., 2023; Beauchamp, 2023). The choice of concentration of tastant was based on published psychophysical data (Hayes et al., 2010; Feeney and Hayes, 2014; Masi et al., 2015), preliminary tests, and a pilot study performed in 10 sensory laboratories with an average number of 5 subjects per lab.

### 2.3 Measurements

#### 2.3.1 Demographic, anthropometric, and general health information

Socio-demographic (gender, age, education) information, self-reported anthropometric data, including weight (kg) and height (m) (that were used to calculate the Body Mass Index, in kg/m^2^), and self-reported problems with taste and smell were collected through the online questionnaire in advance of the test sessions.

#### 2.3.2 Italian Taste-Food Familiarity questionnaire

The original version of the Italian Taste-Food Familiarity questionnaire was designed to measure both the familiarity features of frequency of consumption and levels of product knowledge of a set of 184 food items (Monteleone et al., 2017). Eating scenarios were delineated, encompassing both the conventional Italian meal structure (breakfast, lunch, and dinner) and new trends, such as snacking or lighter meals and aperitifs, which often replace traditional meal timings. The participants were tasked with assessing each food item using a 5-point labeled scale (1 = I do not recognize it; 2 = I recognize it, but I have never tasted it; 3 = I have tasted it, but I don't eat it; 4 = I occasionally eat it; 5 = I regularly eat it (Tuorila et al., 2001)). The present paper used a selection of 88 food items, grouped into 13 food categories. The selection was based on a previously published questionnaire (Predieri et al., 2020). Where applicable, each category was divided based on the food item's energy content (caloric/right energy dense) (Supplementary Table S1).

The sequence in which items appear within each product category, as well as the order of the product categories, is randomized for each participant. The questionnaire was administered online in advance of the test sessions.

#### 2.3.3 Liking and intensity ratings of the food model

The assessment of liking and the intensity of the chosen target sensations - saltiness, umami, and overall flavor - were conducted on two different days. During the first session, participants were prompted to express their overall liking for the four BeanP samples using the Labeled Affective Magnitude Scale (LAM) ranging from 0 to 100 (Schutz and Cardello, 2001). In the subsequent session, individuals evaluated the intensity of the three sensations (saltiness, umami, and overall flavor) for each sample, using the Generalized Labeled Magnitude Scale (gLMS) spanning from 0 to 100 (Bartoshuk et al., 2004). Before tasting, participants followed a training session whereby they were instructed on the use of the scales. In particular, they were directed to utilize the Generalized Labeled Magnitude Scale (gLMS), as described in established guidelines (Bartoshuk et al., 2004; Green et al., 1993; Bartoshuk, 2000).

Subjects received detailed instructions, emphasizing that the "strongest imaginable sensation" should represent the most intense experience they could conceive. To acquaint participants with the scale's endpoints, they were prompted to recall various sensations across different sensory realms, drawn from oral (e.g., the chill of an ice cube in the mouth; the fiery sensation of hot chili pepper) and non-oral experiences (e.g., the sound of a low-flying plane; the pain from accidently shutting a finger in a door). These examples were proposed to stimulate discussion and aid in familiarizing individuals with the scale's extremes.

For each session, the samples, each containing 15 g of BeanP, were served at room temperature in plastic cups labeled with unique three-digit codes. Participants were instructed to consume the entire provided portion before rating their liking or the intensity of sensations. A 90-s interval between tastings was enforced, during which participants were offered still oligomineral water for palate cleansing. The presentation order of the samples was methodically altered following William's Latin square arrangement.

#### 2.3.4 Sensitivity to punishment and sensitivity to reward questionnaire

The *Sensitivity to Punishment and Sensitivity to Reward Questionnaire* (SPSRQ) was used to measure the reactivity of the Behavioral Activation System (BAS) and the Behavioral Inhibition System (BIS) (Torrubia et al., 2001). This questionnaire comprises two scales: the Sensitivity to Punishment scale (SP) assesses individual reactions and responsiveness to BIS-related stimuli, while the Sensitivity to Reward scale (SR) gauges BAS functionality concerning specific rewards (e.g., money, praise, social power). Each scale uses a yes/no format, with the total score per participant calculated by summing the "yes" responses. The original version of the questionnaire provided scores for each scale ranging from 0 to 24, with higher scores reflecting, respectively, higher sensitivity to punishment and reward.

#### 2.3.5 Food neophobia scale

The validated Italian version of the Food Neophobia Scale (FNS; (Pliner and Hobden, 1992)) was used to measure the reluctance to try new and unfamiliar foods (Laureati et al., 2018). The scale comprises 10 items, each rated on a 7-point Likert scale (1 = strongly disagree; 7 = strongly agree). The FNS score, ranging from 10 to 70, is derived by summing the ratings after appropriate reverse scoring. Higher scores indicate greater food neophobia.

### 2.4 Data analysis

The data matrix was properly treated for missing data and checked for normality. Two-hundred and twelve subjects were excluded due to self-declaration of suffering from problems affecting taste and/or smell perception (except cold) or due to several missing data in the responses related to overall liking and perceived intensity of the BeanP samples.

Pearson correlation coefficients were computed between individual liking and salty, umami, and overall flavor intensity scores across the four concentrations of bean purée samples following an approach described in Endrizzi et al. (2021) and Spinelli et al. (2021). Cluster analysis on *r-values* was employed to identify clusters of individuals who exhibited similar sensory-hedonic patterns. Ninety-four subjects were excluded from the cluster analysis due to an inability to calculate their correlation coefficient, primarily due to zero variance in evaluating at least one sensory property. A k-means clustering algorithm was chosen due to its reported computational efficiency and scalability, particularly in handling large datasets (Jain, 2010). Previous data, applying different clustering approaches and considering different variables to clustering, such as hedonic scores (Pangborn, 1970; Proserpio et al., 2025) or hedonic score and salty intensity ratings (Endrizzi et al., 2021), suggested a variable number of clusters from 2 to 4. Thus, a range of number of clusters (from 2 to 5) was considered in the present study. To determine the optimal number of clusters, the Elbow method was applied to minimize the within-cluster inertia, and the silhouette analysis to maximize the silhouette score. The Hartigan index was also used to evaluate the quality of clustering solutions. To ensure robustness, the k-means algorithm was run multiple times with different initial centroid seeds, producing stable results. To confirm the separability of the clusters along the first two components, PCA scatter plots were used for visualization (see Supplementary Materials).

A two-way analysis of variance (ANOVA) model was applied to test the effect of clusters and samples and their interaction on liking and sensory responses (salty, umami, overall flavor intensity) and, thus, verify the different sensory-hedonic patterns of clusters. Type III Sum of Squares and Least Squares means were used and the Bonferroni *post-hoc* test was applied (p ≤ 0.05). The effect size of each factor was determined using partial eta squared (*η*^2^). Small, medium, and large effects correspond to values of partial η^2^ of 0.0099, 0.0588, and 0.1379, respectively (Cohen, 2013). t-tests were applied to characterized clusters by age and BMI, all considered continuous variables. The associations among clusters and gender (women *vs* men), educational level (Primary/secondary education *vs* University/post-university degree), class age (18–30 *vs* 31–45 *vs* 46–60), and nutritional status (lean *vs* overweight/obese) were investigated using chi-square tests. Fisher's exact test was run to test the significance by cell (significance level fixed at α = 0.05).

Data were then analyzed disaggregated by gender and the optimal number of clusters was chosen considering the same criteria reported above. The same statistical models were applied to identify and characterize clusters within each gender.

Cronbach's α was computed for the *Food Neophobia Scale*. The value of 0.70 was set as the lowest acceptable limit for the satisfactory internal consistency of the measure. The scale fulfilled the satisfactory level of internal consistency (α = 0.87). t-tests were used to compare FN scores between clusters within each gender and chi-square tests were used to compare distribution in each food neophobia class (cut-off values: low FNS score ≤18; medium: 18 < FNS score <36; high: FNS score ≥36) as defined in Laureati et al. (2018). Fisher's exact test was run to test the significance by cell (significance level fixed at p = 0.05).

As indicated in the validation of the Italian version of the questionnaires, seven items (4, 8, 16, 25, 32, 24, 26) were not considered for the *Sensitivity to Punishment and Sensitivity to Reward Questionnaire* (Spinelli et al., 2018). Guttman split-half reliability was calculated, due to the binary nature of the data. A coefficient value above 0.70 is typically seen as acceptable for internal reliability. Both scales fulfilled a satisfactory level of internal consistency, reporting respectively 0.85 for SP and 0.71 for SR. The scores ranged from 0 to 23 for SP and 0 to 18 for SR, where higher scores signify greater sensitivity to punishment and reward, respectively. t-tests were used to compare SP and SR scores between clusters within each gender.

For the *Italian Taste-Food Familiarity questionnaire*, a familiarity/frequency of use score (FFS) was computed by summing up the ratings with each food category for each subject, as proposed by Dinnella et al. (2023). t-tests were used to assess the main effects of cluster or nutritional status on FFS of each food category within each gender.

All data analyses were conducted using XLSTAT v2023.April 1, 1408 (Lumivero, Denver, USA).

## 3 Results

### 3.1 Analyses of the whole sample

#### 3.1.1 Clusters’ segmentation

**Fig. 2:**
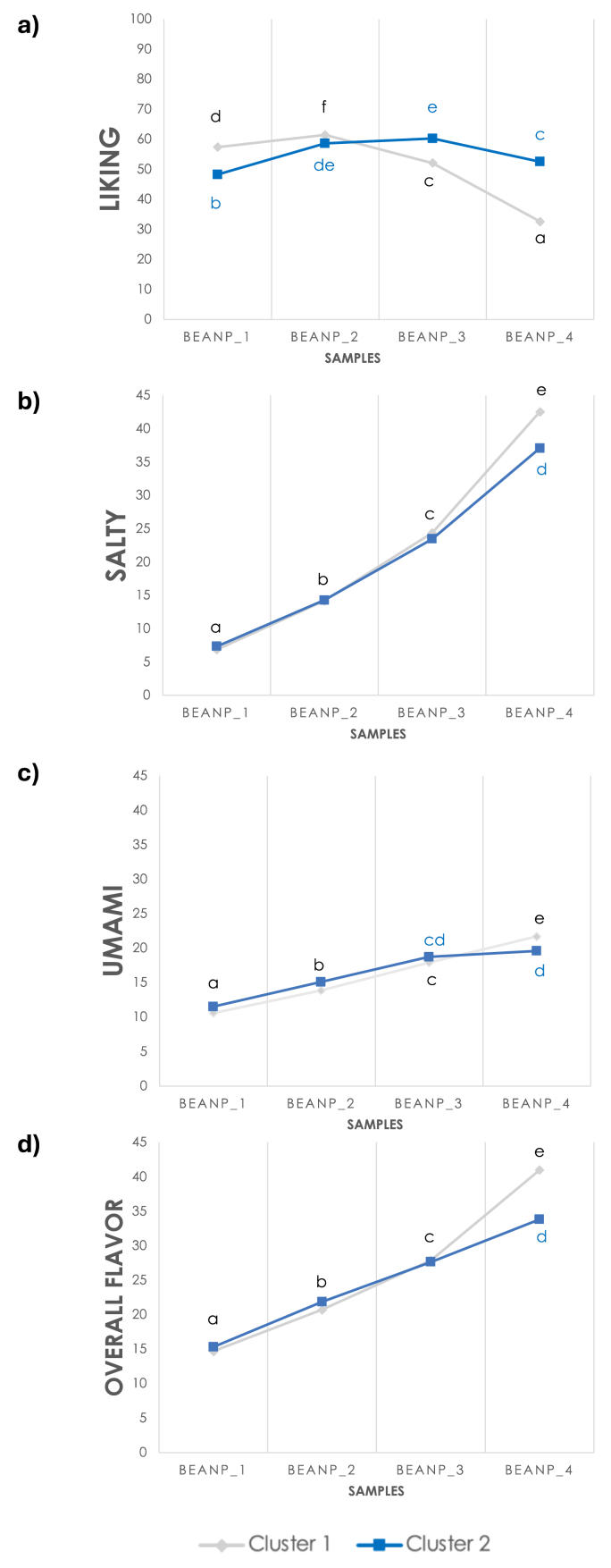
**a-d.** Mean liking (a) and intensity ratings of salty (b) and umami (c) tastes and overall flavor (d) in the two clusters for each sodium chloride concentration in bean purees samples. Different letters indicate significant differences (p ≤ 0.05).

K-means cluster analysis on correlation coefficients considering the whole sample (n = 2878) revealed the identification of two clusters (Supplementary Table S2; Supplementary Figs. S1 and S2). In Cluster 1 (n = 1850, 64.2%), a negative association was found between liking and salty and umami tastes and overall flavor, while a positive correlation existed for Cluster 2 (n = 1028, 35.8%). Two-way analysis of variance revealed significant main effects of *product* (F_(3, 11504)_ = 559.7; p < 0.001; η^2^ = 0.18), *cluster* (F_(1, 11504)_ = 161.2; p < 0.001; η^2^ = 0.01), and the interaction *cluster∗product* (F_(3, 11504)_ = 408.1; p < 0.001; η^2^ = 0.10) on liking. The analysis highlighted that the two clusters exhibited distinct sensory-hedonic patterns: Cluster 1 exhibited a peak at sample BeanP_2 before decreasing at higher concentrations (BeanP_3 and BeanP_4). Cluster 2 included individuals who enjoyed all samples, with liking scores ≥ 50 on gLMS, with a pattern of an inverted-U shape indicating an optimum for samples BeanP_2 and BeanP_3 (Fig. 2a). The model was significant for all sensory properties. A significant effect of *cluster* was found for salty (F_(1, 11504)_ = 31.8; p < 0.001; η^2^ = 0.01) and overall flavor (F_(1, 11504)_ = 31.3; p < 0.001; η^2^ = 0.01). A main effect of *product* and *cluster∗ product* was evident for salty (product: F_(3, 11504)_ = 3033.8; p < 0.001; η^2^ = 0.48; cluster∗product: F_(3, 11504)_ = 28.3; p < 0.001; η^2^ = 0.01) and umami (product: F_(3, 11504)_ = 257.7; p < 0.001; η^2^ = 0.07; cluster∗ product: F_(3, 11504)_ = 8.6; p < 0.001; η^2^ = 0.01) tastes, and overall flavor (product: F_(3, 11504)_ = 1455.3; p < 0.001; η^2^ = 0.31; cluster∗product: F_(3, 11504)_ = 59.9; p < 0.001; η^2^ = 0.02). The intensity ratings for saltiness and overall flavor, and to a lesser extent, umami, displayed a consistent trend across the two clusters: all sensations increased with higher concentrations of sodium chloride. However, Cluster 1 perceived all target sensations with significantly greater intensity in the BeanP_4 sample (the one with the highest concentration in sodium chloride) than Cluster 2 (Fig. 2b–d).

#### 3.1.2 Clusters’ characterization

Table 1 shows the socio-demographic and anthropometric characteristics of the total participants’ sample and the two clusters differing in their sensory-hedonic patterns for savory tastes. The whole sample was well represented for all the age classes and balanced by gender and educational level. Moreover, it presented a higher percentage of normal-weight respondents (67.1%). Significant differences in average age (t = 1.9; p < 0.05), gender distribution (χ^2^ = 16.4; p < 0.0001), and educational level (χ^2^ = 3.8; p < 0.01) were found between the two clusters, with Cluster 1 characterized by a greater percentage of older subjects, women and with a qualification superior to a secondary school diploma. Moreover, significant differences in BMI (t = −2.5; p < 0.01) and nutritional status distribution (χ^2^ = 3.9; p < 0.0001) were observed, with a higher BMI and the percentage of overweight and obese individuals higher in Cluster 2 (inverted U shape) than in Cluster 1 (who dislike salt at higher concentrations).

**Table 1:** Sociodemographic and anthropometric characteristics of total sample size and two clusters. Values in bold are significant at p < 0.05. The < and > indicate that the observed value is significantly lower or higher than the expected theoretical value (Chi-square per cell significant for α = 0.05; Fisher Exact Probability Test).

	Total (n = 2878)	Cluster 1 (n = 1850; 64.1 %)	Cluster 2 (n = 1028; 35.9 %)	p-value
**Socio-demographic characteristics**				
*Age (mean SD)*	38.3 (12.9)	38.7 (12.9)	37.7 (12.9)	**0.05**
*Age class (%)*				
18–30	34.9%	33.7 %	37.1 %	0.19
31–45	30.8%	31.2 %	30.0 %	
46–60	34.3%	35.1 %	32.9 %	
*Gender (%)*				
Women	54.5 %	57.3 % >	49.5 % <	**0.0001**
Men	45.5 %	42.7 % <	50.5 % >	
*Education level (%)*				
Primary/secondary education	53.8 %	52.1 % <	56.9 % >	**0.01**
University/post-university degree	46.2 %	47.9 % >	43.1 % <	

**Anthropometric characteristics**				
*BMI (mean SD)*	23.9 (4.0)	23.8 (4.0)	24.2 (4.0)	**0.01**
*Nutritional status (%)*^a^				
Lean	67.1 %	68.4 % >	64.8 % <	**0.05**
Overweight/Obese	32.9 %	31.6 % <	35.2 % >	

a The classification of nutritional status was obtained according to World Health Organization (WHO) guidelines (World Health Organization, 2000), considering ‘lean’ subjects with a BMI <24.99 kg/m^2^ and ‘overweight/obese’ subjects with a BMI >25 kg/m^2^.

### 3.2 Analyses by gender

#### 3.2.1 Clusters’ segmentation

**Fig. 3:**
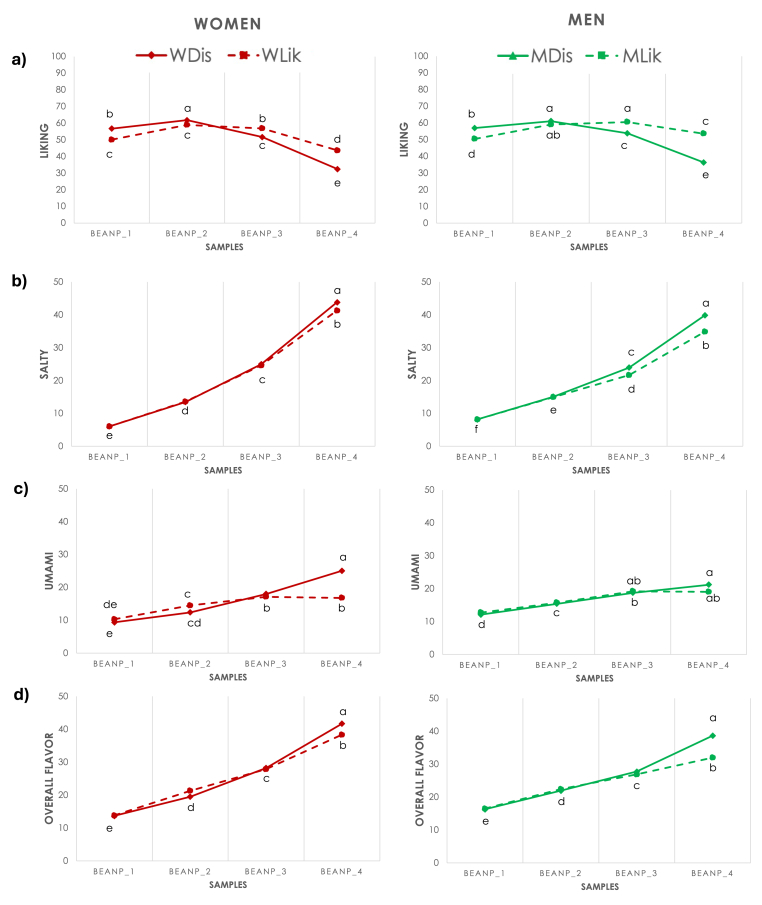
a–d. Mean liking (a) and intensity ratings of salty (b) and umami (c) tastes and overall flavor (d) in each cluster within women (red lines) and men (green lines) groups for each sodium chloride concentration in bean purees samples. Different letters indicate significant differences (p ≤ 0.05).

The same clustering method was used to identify specific sensory-hedonic segments within each gender. K-means cluster analysis on correlation coefficients revealed the identification of two clusters both in women and men groups with similar sensory-hedonic patterns (Supplementary Table S2; Supplementary Figs. S1 and S2): one presenting a negative association between liking and salty and umami tastes and overall flavor, and another a positive correlation (Fig. 3a–d). Two-way analysis of variance revealed significant main effects of *product* (W: F_(3, 6272)_ = 489.2; p < 0.001; η^2^ = 0.19; M: F_(3, 5224)_ = 218.0; p < 0.001; η^2^ = 0.16), *cluster* (W: F_(1, 6272)_ = 14.6; p < 0.001; η^2^ = 0.01; M: F_(3, 5224)_ = 77.2; p < 0.001; η^2^ = 0.02), and the interaction *cluster∗product* (W: F_(1, 6272)_ = 80.1; p < 0.001; η^2^ = 0.04; M: F_(3, 5224)_ = 141.0; p < 0.001; η^2^ = 0.08) in both women and men on liking scores (Fig. 3a). The women group was, indeed, divided into ‘Dislikers’ (WDis; n = 872) and ‘Moderate Likers’ (WLik; n = 698), while men were divided into ‘Dislikers’ (MDis; n = 838) and ‘Likers’ (MLik; n = 470). ‘Dislikers’ in both groups exhibited a peak at sample BeanP_2 before decreasing at higher concentrations (BeanP_3 and BeanP_4). In particular, ‘Likers’ in men group gave higher liking scores for the most concentrated samples (BeanP_3: 60.6 ± 0.5; BeanP_4: 53.6 ± 0.5) compared to women who, for this reason, were labeled as ‘Moderate Likers’ (BeanP_3: 56.8 ± 0.5; BeanP_4: 43.6 ± 0.5). As well, the ‘Dislikers’ in the women group gave the lowest score to the BeanP_4 (32.4 ± 0.5) (Supplementary Fig. S3).

As well, the model was significant for all sensory properties both in women and men (Fig. 3b–d).

As regards to women, a significant effect of *cluster* was found for salty (F_(1, 6272)_ = 4.3; p < 0.05; η^2^ = 0.01) and umami (F_(1, 6272)_ = 19.1; p < 0.001; η^2^ = 0.01) and the main effect of *product* and *cluster∗product* was evident for salty (product: F_(3, 6272)_ = 2127.5; p < 0.001; η^2^ = 0.51; cluster∗product: F_(3, 6272)_ = 3.2; p < 0.05; η^2^ = 0.01), umami (product: F_(3, 6272)_ = 192.7; p < 0.001; η^2^ = 0.09; cluster∗product: F_(3, 6272)_ = 44.4; p < 0.001; η^2^ = 0.02), and overall flavor (product: F_(3, 6272)_ = 1147.8; p < 0.001; η^2^ = 0.36; cluster∗product: F_(3, 6272)_ = 10.4; p < 0.001; η^2^ = 0.01).

As regards to men, a significant effect of *cluster* was found for salty (F_(1, 5224)_ = 25.6; p < 0.001; η^2^ = 0.01) and overall flavor (F_(1, 5224)_ = 22.3; p < 0.001; η^2^ = 0.02) and the main effect of *product* and *cluster∗sample* was evident for salty (sample: F_(3, 5224)_ = 1170.1; p < 0.001; η^2^ = 0.43; cluster∗product: F_(3, 5224)_ = 10.2; p < 0.001; η^2^ = 0.01), umami (product: F_(3, 5224)_ = 86.3; p < 0.001; η^2^ = 0.06; cluster∗product: F_(3, 5224)_ = 3.1; p < 0.05; η^2^ = 0.01), and overall flavor (product: F_(3, 5224)_ = 490.9; p < 0.001; η^2^ = 0.25; cluster∗product: F_(3, 5224)_ = 21.5; p < 0.001; η^2^ = 0.02). Looking at the data together (Supplementary Fig. S4), the greatest difference in terms of perception of the target sensations is particularly evident at higher concentrations, where women 'Dislikers' consistently assign higher intensity scores, and this is especially noticeable for the umami taste (25.1 ± 0.4), while men ‘Likers’ rated lower the intensity of salty taste (34.8 ± 0.6) and overall flavor in sample BeanP_4 (32.0 ± 0.6).

#### 3.2.2 Sensory-hedonic patterns’ characterization

Table 2 shows the socio-demographic and anthropometric characteristics of the taste clusters separated by gender. Significant differences in age (χ^2^ = 9.3; p = 0.009) and educational level (χ^2^ = 6.9; p = 0.008) distributions were observed. In the women group, the ‘Moderate Likers’ presented a greater percentage of subjects from the younger class (18–30 years; 39.8%), while in the men group the ‘Likers’ presented a greater percentage of subjects with a lower educational level (61.7%). In the men group, the ‘Likers’ group was characterized by subjects with a higher BMI (25.2 ± 3.6) than the ‘Dislikers’ (24.9 ± 3.8), although the differences were not significant (p = 0.09).

**Table 2:** Sociodemographic and anthropometric characteristics of taste clusters within each gender group. Values in bold are significant at p < 0.05. The < and > indicate that the observed value is significantly lower or higher than the expected theoretical value (Chi-square per cell significant for α = 0.05; Fisher Exact Probability Test).

	Women			Men		
	**WDis** (n = 872; 55.5 %)	**WLik** (n = 698; 44.4 %)	p	**MDis** (n = 838; 64.1 %)	**MLik** (n = 470; 35.9 %)	p
**Socio-demographic characteristics**						
*Age class (%)*			**0.009**			0.98
18–30	32.8% <	39.8% >		33.5%	34.0%	
31–45	33.4% >	28.0% <		30.7%	30.7%	
46–60	33.8%	32.2%		35.8%	35.3%	
						
*Education level (%)*			0.16			**0.008**
Primary/secondary education	48.9%	52.4%		54.2% <	61.7% >	
University/post-university degree	51.1%	47.6%		45.8% >	38.3% <	

**Anthropometric characteristics**						
*BMI (mean SD)*	22.9 (3.9)	23.0 (4.1)	0.60	24.9 (3.8)	25.2 (3.6)	0.09
*Nutritional status (%)*						
Lean	77.2%	76.9%	0.90	56.3%	53.0%	0.24
Overweight/Obese	22.8%	23.1%		43.7%	47.0%	

The effect of taste cluster was independently tested for women and men on the personality trait scores (Table 3). A tendency, although not significant (p = 0.06), was highlighted in the men group for the FN level, with the ‘Likers’ characterized by subjects with a more pronounced neophobic attitude (30.8%) than Dislikers (25.5%).

**Table 3:** Personality and psycho-attitudinal traits of taste clusters within each gender group. The < and > indicate that the observed value is significantly lower or higher than the expected theoretical value (Chi-square per cell significant for α = 0.05; Fisher Exact Probability Test).

Personality and psychological traits	Women			Men		
	WDis (n = 872; 55.5 %)	WLik (n = 698; 44.5 %)	p-value	MDis (n = 838; 64.1 %)	MLik (n = 470; 35.9 %)	p-value
*Neophobia score (mean SD)*	26.7 (11.8)	26.4 (11.4)	0.40	28.0 (11.0)	28.5 (11.5)	0.27
*Neophobia level (%)*			0.90			0.06
High	25.3%	24.2%		25.5% <	30.8% >	
Medium	45.5%	46.3%		51.9% >	44.1% <	
Low	29.2%	29.5%		22.6%	25.1%	

*Sensitivity to punishment (mean SD)*	9.4 (5.4)	9.3 (5.2)	0.66	9.2 (5.4)	9.1 (5.5)	0.53
*Sensitivity to reward (mean SD)*	5.7 (3.7)	5.6 (3.7)	0.61	6.1 (3.8)	6.2 (3.7)	0.16

No effect was found on *Sensitivity to Punishment/Sensitivity to Reward Questionnaire* subscales for both genders.

### 3.3 Gender- and nutritional status-based differences in FFS

The effect of taste clusters was independently assessed in women and men on FFS (Table 4). In general, ‘Likers’ (irrespective of gender) displayed a significantly higher familiarity/use frequency of specific food categories, such as caloric breakfast products (W: t = −2.49; p = 0.01; M: t = −2.52; p = 0.01), caloric meals dishes/junk foods (W: t = −4.65 p < 0.001; M: t = −3.75; p < 0.001), red and cured meat (W: t = −2.84; p = 0.01; M: t = −3.04; p = 0.002), lean meat (W: t = −3.97; p < 0.001; M: t = −2.08; p = 0.04) and both saturated (W: t = −2.26; p = 0.03; M: t = −3.01; p = 0.003) and unsaturated (W: t = −2.96; p = 0.003; M: t = −2.60; p = 0.009) fats.

**Table 4:** Taste clusters-related differences in familiarity/consumption frequency of food categories within Women and Men groups. Data are reported as mean values (SD). Values in bold are significant at p < 0.05.

Food Category a	Variation in energy content a	Women			Men		
		WDis	WLik	p-value	MDis	MLik	p-value
Fruits (0–20)		17.1 (2.2)	17.3 (1.9)	0.15	17.1 (2.1)	17.2 (1.8)	0.40
Vegetables (0–65)		54.6 (6.7)	55.1 (6.1)	0.17	54.2 (6.8)	54.5 (6.2)	0.42
Legumes (0–25)		20.0 (2.6)	20.2 (2.3)	0.09	19.8 (2.6)	19.9 (2.4)	0.76
Savory snacks (0–20)		14.8 (2.0)	15.0 (2.0)	0.16	14.7 (1.9)	15.1 (1.9)	**0.002**
Breakfast products	Caloric (0–25)	14.8 (2.1)	15.1 (2.2)	**0.01**	14.7 (2.2)	15.0 (2.0)	**0.01**
	Light (0–20)	15.1 (2.1)	15.2 (2.1)	0.10	15.0 (2.1)	15.1 (2.1)	0.573
Caloric meal dishes/junk foods (0–20)		16.0 (1.9)	16.4 (1.8))	**0.001**	16.0 (2.0)	16.4 (1.7)	**0.001**
Cheeses	Light (0–15)	11.4 (2.1)	11.6 (1.9)	0.08	11.2 (2.0)	11.4 (1.8)	0.09
	Caloric (0–30)	22.3 (3.8)	22.6 (3.8)	0.11	22.4 (3.7)	22.7 (3.5)	0.08
Dairy products	Full cream (0–10)	7.5 (1.4)	7.7 (1.4)	0.08	7.6 (1.4)	7.6 (1.3)	0.70
	Skimmed (0–10)	7.6 (1.4)	7.7 (1.4)	0.40	7.6 (1.4)	7.7 (1.4)	0.07
Meat	Red + cured (0–40)	32.1 (4.4)	32.8 (4.0)	**0.01**	32.1 (4.4)	32.9 (3.9)	**0.002**
	Lean (0–15)	12.1 (1.8)	12.5 (1.6)	**0.001**	12.1 (1.8)	12.3 (1.6)	**0.04**
Seafood (0–35)		26.8 (3.8)	27.3 (3.7)	**0.01**	26.7 (4.0)	26.9 (3.6)	0.41
Beverages	Alcoholic beverages (0–20)	15.1 (2.8)	15.2 (2.6)	0.42	15.4 (1.8)	15.5 (2.7)	0.85
	Soft drinks (0–15)	11.1 (1.7)	11.2 (1.7)	0.17	11.0 (1.8)	11.2 (1.7)	**0.04**
Fats	Saturated (0–10)	7.1 (1.1)	7.2 (1.1)	**0.03**	7.0 (1.1)	7.2 (1.1)	**0.003**
	Unsaturated (0–15)	12.5 (1.6)	12.8 (1.3)	**0.003**	12.6 (1.5)	12.8 (1.3)	**0.009**
Sweets/Desserts (0–30)		22.8 (2.2)	23.1 (2.2)	**0.01**	22.6 (2.7)	22.9 (2.5)	0.06

a Values in brackets correspond to the minimum-maximum values of FFS in each food category.

Gender-related differences were also highlighted, with ‘Likers’ in the women group reporting a higher familiarity/use frequency of seafood (t = −2.58; p = 0.01) and desserts (t = −2.68; p = 0.01) and men ‘Likers’ reporting a higher familiarity/use frequency of savory snacks (t = −3.1; p = 0.002) and soft drinks (t = −2.02; p = 0.04).

The effect of nutritional status was independently assessed in women and men on familiarity scores used to evaluate their food consumption habits (Table 5). As expected, overweight and obese subjects (irrespective of gender) generally demonstrated less healthy food behaviors, displaying higher consumption of caloric meals/junk foods (W: t = −2.45; p = 0.01; M: t = −2.73; p = 0.006), red and cured meat (W: t = −3.09; p = 0.002; M: t = −3.13; p = 0.002) and saturated fats (W: t = −2.48; p = 0.01; M: t = −2.35; p = 0.02). Moreover, gender-related differences were highlighted, with men characterized by overweight/obese status reporting also a higher consumption frequency of savory snacks (t = −2.49; p = 0.01), caloric breakfast (t = −1.96; p = 0.05), and lean meat (t = −2.56; p = 0.01).

**Table 5:** Nutritional status-related differences in familiarity/consumption frequency of food categories within Women and Men groups. Values in bold are significant at p < 0.05. Data are reported as mean values (SD).

Food Category ^a^	Variation in energy content a	Women			Men		
		Lean	Overweight/Obese	p-value	Lean	Overweight/Obese	p-value
Fruits (0–20)		17.2 (1.9)	17.3 (2.2)	0.34	17.1 (2.1)	17.2 (1.9)	0.35
Vegetables (0–65)		54.8 (6.2)	54.9 (7.0)	0.89	54.2 (6.6)	54.5 (6.5)	0.47
Legumes (0–25)		20.0 (2.4)	20.2 (2.7)	0.39	19.8 (2.6)	19.9 (2.5)	0.29
Savory snacks (0–20)		14.8 (1.9)	15.0 (2.3)	0.16	14.7 (2.1)	15.0 (1.9)	**0.01**
Breakfast products	Caloric (0–25)	14.9 (2.1)	15.0 (2.2)	0.39	14.7 (2.2)	15.0 (2.1)	**0.05**
	Light (0–20)	15.2 (2.0)	15.1 (2.3)	0.46	15.0 (2.2)	15.1 (2.1)	0.781
Caloric meal dishes/junk foods (0–20)		16.1 (1.8)	16.4 (2.1)	**0.01**	16.0 (1.9)	16.3 (1.9)	**0.006**
Cheeses	Light (0–15)	11.4 (1.9)	11.6 (2.1)	0.25	11.2 (1.9)	11.4 (1.9)	0.10
	Caloric (0–30)	22.4 (3.7)	22.5 (3.8)	0.74	22.5 (3.6)	22.6 (3.6)	0.69
Dairy products	Full cream (0–10)	7.6 (1.4)	7.6 (1.4)	0.98	7.6 (1.4)	7.6 (1.3)	0.88
	Skimmed (0–10)	7.7 (1.4)	7.6 (1.4)	0.61	7.6 (1.4)	7.7 (1.4)	0.11
Meat	Red + cured (0–40)	32.2 (4.1)	33.0 (4.4)	**0.002**	32.0 (4.4)	32.8 (3.9)	**0.002**
	Lean (0–15)	12.3 (1.7)	12.4 (1.7)	0.16	12.0 (1.8)	12.3 (1.7)	**0.01**
Seafood (0–35)		27.0 (3.7)	26.9 (4.2)	0.66	26.6 (3.9)	27.0 (3.8)	0.14
Beverages	Alcoholic beverages (0–20)	15.3 (2.6)	14.9 (2.9)	0.06	15.4 (2.8)	15.5 (2.5)	0.93
	Soft drinks (0–15)	11.1 (1.7)	11.2 (1.7)	0.25	11.0 (1.8)	11.2 (1.7)	0.11
Fats	Saturated (0–10)	7.1 (1.1)	7.3 (1.2)	**0.01**	7.0 (1.1)	7.2 (1.7)	**0.02**
	Unsaturated (0–15)	12.6 (1.4)	12.7 (1.7)	0.53	12.6 (1.5)	12.7 (1.4)	0.18
Sweets/Desserts (0–30)		22.9 (2.1)	22.8 (2.3)	0.52	22.7 (2.7)	22.8 (2.6)	0.56

a Values in brackets correspond to the minimum-maximum values of FFS in each food category.

## 4 Discussion

The present study focused on examining sensory-hedonic patterns for both umami and salty tastes in a large sample of adult Italian consumers. Considering the whole sample of consumers, the results of the present study suggested that a sensory-hedonic pattern characterized by lower perceived intensity for saltiness and umami and a greater preference for high concentrations of salty stimulus (i.e., Cluster 2) seem to be more strongly associated with male subjects and with unhealthy anthropometric parameters. As far as salt taste is regarded, the present findings are in line with the hypothesis that a positive correlation between higher BMI and increased preference for sodium chloride exists (Li et al., 2018), and with the general assumption that inter-individual differences in responsiveness to salty taste (i.e., reduced) characterized individuals with overweight or obesity condition (Li et al., 2018; Coltell et al., 2019; Harnischfeger and Dando, 2021; Veček et al., 2020; Cattaneo et al., 2023). The potential link between high monosodium glutamate consumption and overweight/obesity remains to be clarified, as the findings in the literature are still controversial (Andres-Hernando et al., 2021; He et al., 2008; Zhang et al., 2020; Yeomans, 2023; Sasano et al., 2014; Hermanussen and Tresguerres, 2003). Future long-term studies exploring the association between taste patterns for ‘savory stimuli’ and the risk of chronic diseases, such as obesity, are needed for a comprehensive understanding of whether different sensory-hedonic patterns can serve as a valuable screening tool for predicting risk. Moreover, additional assessments, such as body composition analysis or fat distribution measures, should be considered to obtain a broader picture of the individual nutritional status, since the BMI as a standalone health indicator has come under scrutiny in recent years (Gutin, 2021; Fayyaz et al., 2024).

The cluster segmentation on the whole sample obtained in the present work closely aligns with the two clusters identified in the preliminary study by Proserpio et al. (2025), which was based solely on liking data, and highlights the same general trends of the sensory-hedonic patterns identified by Endrizzi and colleagues (Endrizzi et al., 2021). In fact, the two current clusters merge the previous four into negative and positive correlations between liking and intensity scores. Cluster 1, for which liking decreases with increasing salt concentration, is characterized by a higher percentage of women, supporting the idea that women tend to find higher levels of salty and umami tastes less pleasant than men (Hayes et al., 2010; Cecchini et al., 2019). Since it is widely reported in the literature that gender and gender-specific attitudes and motivations significantly influence sensory-hedonic patterns, eating behaviors, as well as individual responses to dietary intake (Endrizzi et al., 2021; Hayes et al., 2010; Spinelli et al., 2020; 2021; Puputti et al., 2019; Li et al., 2012; Dinnella et al., 2023; Teleman et al., 2016), a within-gender approach was applied in the present study. Thus, women and men were categorized independently into two groups: the women group was divided into ‘Dislikers’ And ‘Moderate-Likers’, while men were divided into ‘Dislikers’ and ‘Likers’. The trend of liking scores is similar in both genders: 'Dislikers' exhibit a decrease in liking scores as the salt concentration increases in the more concentrated samples (BeanP_3 and BeanP_4), whereas 'Likers' achieve their optimum liking at a higher salt concentration (BeanP_3) (Fig. 3a). The primary difference between men and women lies in their liking scores for the most concentrated sample. Men in both clusters significantly prefer it more than women, who consistently rate it as the least liked. For this reason, women have been defined as 'Moderate Likers,' as they generally appear to prefer lower salt concentrations in the product (Supplementary Fig. S3). Regarding taste responsiveness, generally 'Likers' rated the stimuli at higher concentrations of sodium chloride as less intense in saltiness, umami, and overall flavor, compared to 'Dislikers', although this association was less pronounced in men. In the women group, the ‘Moderate-Likers’ showed a higher proportion of subjects in the younger age bracket (18–30; 39.8%). Conversely, in the men group, the ‘Likers’ exhibited a higher percentage of subjects with a lower level of education (61.7%) and were characterized by a general overweight condition (mean BMI value > 25 kg/m^2^). These latter results supported the outcome already widely debated in the literature about the positive relation between lower educational level, higher consumption of palatable foods, and overweight/obesity conditions (Stival et al., 2022).

Contrary to our hypothesis, no difference in personality and psychological traits between clusters within each gender was found. One might have expected that higher sensitivity to reward could characterize the taste patterns inclined toward these stimuli (i.e., ‘Moderate-Likers’ and ‘Likers’), because of being more prone to the reward effect associated with palatable and appetizing foods. However, the present findings failed to highlight this association. Thus, in the absence of further scientific data to support/disconfirm these findings, future research is needed to explore this aspect more comprehensively, including a broader range of variables related to individual personality traits, such as orientations toward the health and hedonic characteristics of foods, and eating behaviors in response to emotional and external factors, and restrained eating. To mention, a tendency, although not significant, was highlighted in the group of men for the food neophobia trait, with men ‘Likers’ characterized by a more pronounced neophobic attitude (30.8%). Overall, these results seemed to highlight a particularly interesting gustatory pattern (i.e., men ‘Likers’) that requires special attention in analyzing the contributing factors to healthy/unhealthy eating behavior. In this group, a preference for foods characterized by satisfying tastes such as salty and umami, their related lower taste sensitivity, and a possibly more pronounced neophobic attitude may contribute to a higher body mass index (Proserpio et al., 2018; Knaapila et al., 2015).

Due to the possible relevance of the taste phenotypes in food habits (i.e., the positive correlations between sweet liker status and liking of sweet foods), we sought to determine if there were associations between different taste patterns for salty/umami stimuli and food habits. Indeed, preliminary data from our research group (Proserpio et al., 2025), based on a sample of about 400 subjects, revealed that clusters with different levels of liking for saltiness, evaluated using the same food model as in the current study, showed differences in food choices. Specifically, the 'Salt Likers' cluster preferred less healthy options, including both savory and non-savory food products that are fattier and more caloric. Consistently herein, both the ‘Moderate-Likers’ and ‘Likers’ (regardless of gender) generally exhibited a notably higher familiarity/use frequency for particular food categories, including high-calorie breakfast items, caloric meal dishes/junk foods, meat, and both saturated and unsaturated fats, which can be generally associated to less healthy eating habits. Although no other study in the literature has specifically focused on taste clusters comparable to those in the present study which encompassed both salty and umami tastes, the main results appear to be comparable to the investigations that have independently explored the relationship between umami or salty taste sensitivity/liking and dietary preferences/consumption habits. Kubota et al. (2018) reported that umami tasters (i.e., people with greater responsiveness to umami) preferred shellfish, tomato, carrot, milk, low-fat milk, cheese, dried shiitake, and kombu significantly more than umami hypo-tasters. Meanwhile, reduced umami taste sensitivity correlated with decreased adherence to the Mediterranean diet in elderly people (Fluitman et al., 2021) and lower consumption of healthy foods, such as vegetables in the Finnish population (Puputti et al., 2019). Kim and Lee (2009) identified a correlation between individuals' sensitivity to salty taste and their acceptance and consumption of sodium-rich fast foods among Korean adolescents. Similarly, Cattaneo et al. (2019) observed that reduced sensitivity to salty taste appears to lead to increased consumption of less healthy foods, such as products rich in saturated fats and soft drinks. Nonetheless, preference for umami and salty are reported to influence food intake (e.g., increased consumption of fast foods or dishes contributing to sodium intake) (Kim and Lee, 2009; Törnwall et al., 2014; Shim et al., 2016). Moreover, gender-related differences were also highlighted, with ‘Moderate-Likers’ in the women group reporting a higher familiarity/use frequency of seafood and desserts and men ‘Likers’ reporting a higher familiarity/use frequency of savory snacks and soft drinks, indicating that the association between salt liking and unhealthier food preference differs according to gender. These results require further confirmation, as the underlying reasons for these gender differences remain unclear. A possible hypothesis could be the differing social pressures women face regarding eating habits and body image (Mallaram et al., 2023), as well as the strong relationship between the consumption of specific food categories and emotional state (Ljubičić et al., 2023). Nevertheless, this taste clusterization appears to play a role in specific dietary patterns, underscoring the importance of studying eating habits from a taste perspective – and not only a nutritional perspective, alongside gender-related variations. However, future studies must consider neurophysiological factors to better understand the observed differences between men and women. It is essential to explore how men and women process sensory inputs like taste or flavor in the brain, the role of hormones (e.g., estrogen, testosterone) in modulating neural responses to stimuli (Schroeder, 2010), and how social or emotional factors related to preferences may differ between genders (Chen and Antonelli, 2020).

Regarding differences in dietary patterns based on weight status, as expected, overweight and obese individuals (regardless of gender) generally exhibited less healthy eating habits. They showed greater familiarity with/more frequent consumption of high-calorie meals, junk foods, red and cured meats, and saturated fats. (Denoth et al., 2016). Moreover, gender-related differences were highlighted, with men characterized by overweight/obese status reporting a higher familiarity/use frequency of salty snacks, light cheeses, and lean meat, in accordance with another population study conducted in the Netherlands which found men with obesity consuming a significantly larger percentage of energy from foods tasting ‘salt, umami and fat’ (van et al., 2018). Thus, gender-tailoring strategies should be taken into account to effectively promote or discourage the consumption of specific food categories in a targeted population.

## 5 Strengths and limitations

The large ethnically homogeneous adult sample, balanced by gender and age ensured the representativeness of the results and allowed the exploration of differences in socio-demographic and anthropometric variables between the cluster obtained, and the application of a within-gender approach. Furthermore, we identified homogeneous groups based on sensory-hedonic responses to savory stimuli, encompassing both umami and salty tastes, enabling the identification of various response patterns among participants. These distinct response patterns led to different food behaviors. However, it is important to exercise caution when applying our findings to individuals of diverse ethnicities or cultures, as identified taste segments may differ among populations. Moreover, it is desirable to consider other food models and define a more generalizable and consistent phenotyping method to identify different savory-hedonic patterns, as previously done with sweet taste phenotypes, thus facilitating the comparisons across different cultures and future studies. An additional limitation included the self-reported anthropometric information used to categorize participants into different BMI groups. Combining BMI with additional assessments, such as body composition analysis, fat distribution measures, and clinical evaluations, would have provided a more accurate and holistic understanding of individuals’ nutritional status. Similarly, the use of self-reported familiarity/consumption frequency data presented a limitation in assessing actual intake. The use of a more quantitative instrument (e.g., a weekly record) could have provided a more accurate evaluation of real food consumption.

## 6 Conclusions

In conclusion, these results suggested the existence of specific taste clusters and underscored the significance of identifying and mapping individual sensory-hedonic patterns to savory stimuli within the two gender groups as possible explanatory variables affecting healthy/unhealthy eating habits. Thus, a sensory-based and consumer-led approach emerges as a promising avenue for probing the intricate nuances of taste perception and its ramifications on food preferences and consumption behaviors. Moreover, these findings pointed out the importance of a within-gender approach to developing more effective strategies in encouraging or discouraging the consumption of specific food categories in specific target groups, holding significant implications for public health interventions aimed at promoting healthier dietary choices among both genders and mitigating the burden of chronic diseases. Appropriate studies and approaches (i.e., structural equation modeling) are needed to quantify and further show the possible causative effects of the complex variable system investigated here. By elucidating the intricate mechanisms linking hedonic/sensory perception to dietary behaviors and health outcomes, researchers can inform targeted interventions tailored to individuals' unique sensory profiles and preferences, thereby fostering more effective strategies for promoting optimal health and well-being.

## CRediT authorship contribution statement

**Camilla Cattaneo:** Conceptualization, Formal analysis, Visualization, Writing – original draft. **Sara Spinelli:** Conceptualization, Methodology, Writing – review & editing. **Caterina Dinnella:** Conceptualization, Methodology, Writing – review & editing. **Cristina Proserpio:** Conceptualization, Writing – review & editing. **Erminio Monteleone:** Conceptualization, Methodology, Writing – review & editing. **Ella Pagliarini:** Conceptualization, Methodology, Writing – review & editing. **Monica Laureati:** Conceptualization, Methodology, Supervision, Writing – review & editing.

## Ethical statement

Ethical approval for the involvement of human subjects in this study was granted by the Ethical Committee of Trieste University (n. 64, June 9, 2015)

## Declaration of generative AI and AI-assisted technologies in the writing process

During the preparation of this work, the author(s) used Chat GPT in order to improve the readability and language of the manuscript. After using this tool/service, the author(s) reviewed and edited the content as needed and take(s) full responsibility for the content of the published article.

## Funding

Each Italian Taste research unit provided the funding for its experimental activity. C.C. and M.L. also acknowledge the project funding under the National Recovery and Resilience Plan (NRRP), Mission 4 Component 2 Investment 1.3-Call for tender No. 341 of March 15, 2022 of Italian Ministry of University and Research funded by the 10.13039/501100000780European Union - NextGenerationEU; Award Number: Project code PE00000003, Concession Decree No. 1550 of October 11, 2022 adopted by the Italian Ministry of University and Research, CUP D93C22000890001, Project title ON Foods-Research and innovation network on Food and Nutrition Sustainability, Safety and Security-Working ON Foods.

## Declaration of competing interest

The authors declare that they have no known competing financial interests or personal relationships that could have appeared to influence the work reported in this paper.

## Data Availability

The data that has been used is confidential.
