# Supplementary material for: Patterns of sensory and hedonic responses for salty and umami tastes and their impact on food familiarity, consumption, and nutritional status: A gender-based analysis from a large population sample
Source: Curr Res Food Sci. 2025 Jan 6;10:100970. doi: 10.1016/j.crfs.2025.100970 (PMC11773254; doi:10.1016/j.crfs.2025.100970)
Supplement: Multimedia component 1 [file mmc1.docx]

**Supplementary material**

**Supplementary Table S1.** List of food items considered in each food category. Each category takes into consideration the variation in taste and/or energy content of food items.

| **Food Category** | **Variation energy content (where applicable)** | **Food items** |
| --- | --- | --- |
| Fruits |  | Apple (Golden Delicious) |
|  |  | Apple (Granny Smith) |
|  |  | Pear |
|  |  | Tangerine |
| Vegetables |  | Carrots  Lettuce  Tomatoes  Zucchini  Asparagus  Broccoli  Cauliflower  Chicory  Cucumber  Fennel  Radish  Rocket  Spinach |
| Legumes |  | Beans |
|  |  | Corn |
|  |  | Green beans |
|  |  | Peas |
|  |  | Soybean sprouts |
| Salty/Savory snacks |  | Chips  Chips with paprika  Green olives  Stuffed olives |
| Breakfast products | Light | Sweet-meal biscuits  Melba toast with jam  Cereals bar  Corn flakes  Granola with dried red berries |
|  | Caloric | Butter cookies  Croissant  Stuffed croissant  Sweet snacks/sponge cake |
| Caloric meal dishes / junk foods |  | Pizza  French fries  Hamburger  Schnitzel |
| Cheeses | Light | Cottage cheese  Mozzarella  Ricotta cheese |
|  | Caloric | Stracchino cheese  Pecorino  Sweet provolone cheese  Gorgonzola cheese  Pecorino (aged)  Hot provolone cheese |
| Dairy | Full cream | Milk  Yoghurt |
|  | Skimmed | Milk  Yoghurt |
| Meat | Lean | Chicken breast  Grilled veal  Roast beef |
|  | Red / cured | Bacon  Beefsteak  Cured ham  Mortadella  Pork chop  Ribs  Salami  Sausages |
| Seafood |  | Anchovies  Cod  Codfish  Salmon  Shrimps  Sole  Tuna |
| Beverages | Alcoholic | Beer  White wine  Red wine  Dry spumante |
|  | Soft drinks | Carbonated drinks  Lemon tea  Peach tea |
| Fats | Unsatured | Extra virgin olive oil  Olive oil  Seeds oil |
|  | Saturated | Butter  Margarine |
| Sweets / Desserts |  | Panna cotta  Tiramisù  Chocolate ice-cream  Milk chocolate  Dark chocolate  Dark chocolate pudding |

**Supplementary Table S2.** Values of the Hartigan (H) and Silhouette indices and the difference between the index of a clustering with k clusters and a clustering with (k-1) clusters of the total sample, women, and men. The bolded value representing the largest difference suggests that 2 clusters should be created (Lumivero, 2024).

|  |  | Number of clusters | | | |
| --- | --- | --- | --- | --- | --- |
|  | Indices | 2 | 3 | 4 | 5 |
| Total sample | Silhouette index | 0.45 | 0.38 | 0.35 | 0.32 |
|  | Hartigan index (H) | **1154.40** | 646.20 | 473.45 | 434.14 |
|  | H(k-1) - H(k) | **1496.88** | 508.20 | 172.75 | 39.31 |
| Women | Silhouette index | 0.48 | 0.35 | 0.36 | 0.32 |
|  | Hartigan index (H) | **755.99** | 515.91 | 203.34 | 226.89 |
|  | H(k-1) - H(k) | **468.11** | 240.08 | 312.56 | -23.55 |
| Men | Silhouette index | 0.42 | 0.35 | 0.33 | 0.27 |
|  | Hartigan index (H) | **503.10** | 248.68 | 176.33 | 192.94 |
|  | H(k-1) - H(k) | **653.41** | 254.42 | 72.34 | -16.60 |

**Supplementary Figure S1.** The Elbow plots illustrate the relationship between the number of clusters and metrics such as explained variance or intra-cluster distance for a) the total sample and both for b) women and c) men groups.

The plots indicate that beyond two clusters, additional clusters yield only marginal improvements in explained variance, supporting the conclusion that two is the optimal number of clusters.

**Supplementary Figure S2.** Visualization of the clusters using PCA scatter plots along the first two components for a) the total sample and both for b) women and c) men groups.

To further support the decision on the selection of the number of clusters we applied Principal Component Analysis on the same date used for the K-means to compare the 2-cluster with the 3-cluster solution (Naes, Varela and Berget, 2018). Figure clearly shows that the solutions with two clusters separate better the clusters along the first component compared to the one with three clusters.

Næs, T., Varela, P., & Berget, I. (2018). Individual differences in sensory and consumer science: Experimentation, analysis and interpretation. Woodhead Publishing.


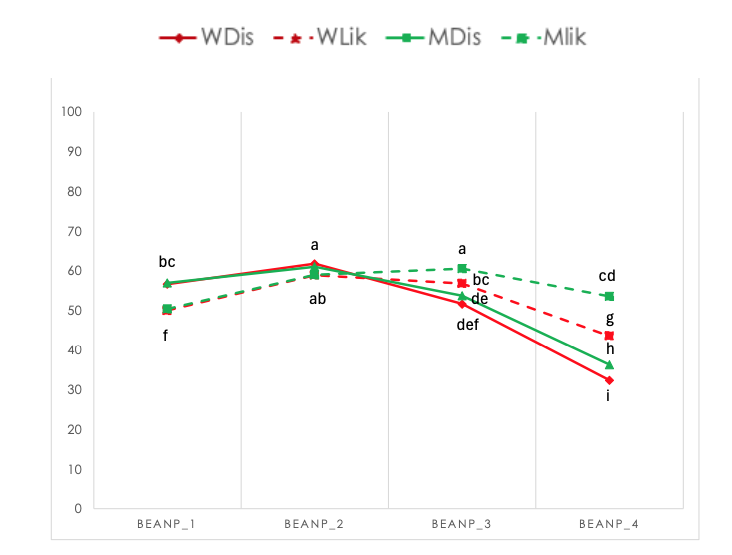


**Supplementary Figure S3.** Mean liking scores in the two taste clusters of each gender for each sodium chloride concentration in bean purees samples. Different letters indicate significant differences (p ≤ 0.05).

**Supplementary Figure S4.** Mean intensity ratings in the two taste clusters of each gender for each sodium chloride concentration in bean purees samples. Different letters indicate significant differences (p ≤ 0.05).
